# Incidence of *Propionibacterium acnes* infection in orthopedic and trauma surgery

**DOI:** 10.1186/2047-2994-4-S1-O28

**Published:** 2015-06-16

**Authors:** I Uckay, A Agostinho, C Landelle, E Coppens, G Cunningham, D Pittet

**Affiliations:** 1 Geneva University Hospitals, Geneva University Hospitals, Geneva, Switzerland; 2 Infection Control Program, Geneva University Hospitals, Geneva, Switzerland

## Introduction

*Propionibacterium acnes* has been associated with late, smoldering and healthcare-associated infections of the shoulder and spine.

## Objectives

The epidemiology of *P. acnes* with respect of other orthopedic locations and patient populations remains largely unknown.

## Methods

Retrospective, single-center, descriptive and case-control studies of adult patients hospitalized for orthopedic infections from 2004-2014. We used only intraoperative microbiological samples and first clinical infection episodes. Cefuroxime (or vancomycine) was used for perioperative prophylaxis. Microbiological samples were incubated for a median of 5 days.

## Results

*P. acnes* was isolated intraoperatively in only 37/2740 (1.35%) surgical procedures. A total of 22/37 infections were monomicrobial. Overall, 665 surgical procedures (24%) involved hardware/osteosynthesis material. *P. acnes* was more frequently identified during procedures in the presence compared with the absence (24/665 vs. 13/2075; *p*<0.01) of hardware/foreign material. *P. acnes* was frequently associated with other skin commensals (12/291 vs. 25/2134; *p*<0.01) and involved the lumbar and shoulder regions. The proportion of *P. acnes* among all pathogens in the spine and shoulder were 8% and 6%, respectively. In contrast, *P. acnes* was almost never identified (3/1021 vs. 334/1719; *p*<0.01) among immune-suppressed patients, in foot infections, septic bursitis, native bone and joint infections, soft tissue abscesses, prosthetic joints, and tibia nails. By multivariate analysis adjusting for case-mix, the lumbar region (odds ratio 7.4, 95% CI 1.2-46.3), the shoulder (OR 9.9, 1.6-60.1) and the presence of hardware (OR 8.2, 2.4-28.4) were significantly associated with *P. acnes* infection; while sex, age, immune-suppression and the administration of antibiotic therapy prior to intraoperative sampling were not.

## Conclusion

In our institution, *P. acnes* is very rarely associated with clinical orthopedic infections. It is almost never responsible for infection below the lumbar spine level. *P. acnes* infections are associated with less inflammatory response than other infections. *P. acnes* is particularly associated with plate and spondylodesis infections.

## Disclosure of interest

None declared.

